# The Evolution of Norms

**DOI:** 10.1371/journal.pbio.0030194

**Published:** 2005-06-14

**Authors:** Paul R Ehrlich, Simon A Levin

## Abstract

Biologists and social scientists need one another, and must collectively direct more of their attention to understanding how social norms develop and change.

Over the past century and a half, we have made enormous progress in assembling a coherent picture of genetic evolution—that is, changes in the pools of genetic information possessed by populations, the genetic differentiation of populations (speciation) (see summaries in [1,2]), and the application of that understanding to the physical evolution of Homo sapiens and its forebears ([3]; e.g., [4,5]). But human beings, in addition to being products of biological evolution, are—vastly more than any other organisms—also products of a process of “cultural evolution.” Cultural evolution consists of changes in the nongenetic information stored in brains, stories, songs, books, computer disks, and the like. Despite some important first steps, no integrated picture of the process of cultural evolution that has the explanatory power of the theory of genetic evolution has yet emerged.

Much of the effort to examine cultural evolution has focused on interactions of the genetic and cultural processes (e.g., [6], see also references in [7]). This focus, however, provides a sometimes misleading perspective, since most of the behavior of our species that is of interest to policy makers is a product of the portion of cultural evolution [8] that occurs so rapidly that genetic change is irrelevant. There is a long-recognized need both to understand the process of human cultural evolution per se and to find ways of altering its course (an operation in which institutions as diverse as schools, prisons, and governments have long been engaged). In a world threatened by weapons of mass destruction and escalating environmental deterioration, the need to change our behavior to avoid a global collapse [9] has become urgent. A clear understanding of how cultural changes interact with individual actions is central to informing democratically and humanely guided efforts to influence cultural evolution. While most of the effort to understand that evolution has come from the social sciences, biologists have also struggled with the issue (e.g., p. 285 of [10], [11–16], and p. 62 of [17]). We argue that biologists and social scientists need one another and must collectively direct more of their attention to understanding how social norms develop and change. Therefore, we offer this review of the challenge in order to emphasize its multidisciplinary dimensions and thereby to recruit a broader mixture of scientists into a more integrated effort to develop a theory of change in social norms—and, eventually, cultural evolution as a whole.

## What Are the Relevant Units of Culture?

Norms (within this paper understood to include conventions or customs) are representative or typical patterns and rules of behavior in a human group [18], often supported by legal or other sanctions. Those sanctions, norms in themselves, have been called “metanorms” when failure to enforce them is punished [17,19,20]. In our (liberal) usage, norms are standard or ideal behaviors “typical” of groups. Whether these indeed represent the average behaviors of individuals in the groups is an open question, and depends on levels of conformity. Conformity or nonconformity with these norms are attributes of individuals, and, of course, heterogeneity in those attributes is important to how norms evolve. Norms and metanorms provide a cultural “stickiness” (p. 10 of [21]) or viscosity that can help sustain adaptive behavior and retard detrimental changes, but that equally can inhibit the introduction and spread of beneficial ones. It is in altering normative attitudes that changes can be implemented.

Here, we review the daunting problem of understanding how norms change, discuss some basic issues, argue that progress will depend on the development of a comprehensive quantitative theory of the initiation and spread of norms (and ultimately all elements of culture), and introduce some preliminary models that examine the spread of norms in space or on social networks. Most models of complex systems are meant to extract signal from noise, suppressing extraneous detail and thereby allowing an examination of the influence of the dominant forces that drive the dynamics of pattern and process. To this end, models necessarily introduce some extreme simplifying assumptions.

Early attempts to model cultural evolution have searched for parallels of the population genetic models used to analyze genetic evolution. A popular analogy, both tempting and facile, has been that there are cultural analogues of genes, termed “memes” [22,23], which function as replicable cultural units. Memes can be ideas, behaviors, patterns, units of information, and so on. But the differences between genes and memes makes the analogy inappropriate, and “memetics” has not led to real understanding of cultural evolution. Genes are relatively stable, mutating rarely, and those changes that do occur usually result in nonfunctional products. In contrast, memes are extremely mutable, often transforming considerably with each transmission. Among humans, genes can only pass unidirectionally from one generation to the next (vertically), normally through intimate contact. But ideas (or “memes”) now regularly pass between individuals distant from each other in space and time, within generations, and even backwards through generations. Through mass media or the Internet, a single individual can influence millions of others within a very short period of time. Individuals have no choice in what genes they incorporate into their store of genetic information, and the storage is permanent. But we are constantly filtering what will be added to our stored cultural information, and our filters even differentiate according to the way the same idea is presented [24,25]. People often deliberately reduce the store of data (for example, when computer disks are erased, old books and reprints discarded, etc.), or do so involuntarily, as when unreinforced names or telephone numbers are dropped from memory. Such qualitative differences, among others, ensure that simple models of cultural evolution based on the analogy to genetic evolution will fail to capture a great deal of the relevant dynamics. A model framework addressed to the specific challenges of cultural evolution is needed.

In the models discussed below, the most basic assumption is that the spread (or not) of norms shares important characteristics with epidemic diseases. In particular, as with diseases, norms spread horizontally and obliquely [14], as well as vertically, through infectious transfer mediated by webs of contact and influence. As with infectious diseases, norms may wax and wane, just as the popularity of norms is subject to sudden transitions [3]. On the other hand, there are unique features of cultural transmission not adequately captured by disease models, in particular the issue of “self-constructed” knowledge, which has long been a source of interest, and the development of problem-solving models in psychology ([26, 27]; D. Prentice, personal communication). New syntheses are clearly required.

## **Microscopic Dynamic**

Substantial progress has been made toward the development of a mathematical theory of cultural transmission, most notably by Cavalli-Sforza and Feldman [14], and Boyd and Richerson [11]. Cavalli-Sforza and Feldman consider the interplay between heritable genetic change and cultural change. This is an important question, addressed to the longer time scale, with a view to understanding the genetic evolution of characteristics that predispose individuals to act in certain ways in specified situations. For many of the phenomena of interest, however, individual behaviors have not evolved specifically within the limited context of a single kind of challenge, but in response to a much more general class of problems. Efforts to provide genetic evolutionary explanations for human decisions today within the narrow contexts in which they occur may be frustrated because generalized responses to evolutionary forces in the distant past have lost optimality, or even adaptive value. Extant human behaviors for example may be the relics of adaptations to conditions in the distant past, when populations were smaller and technology less advanced. Attempts to understand them as adaptive in current contexts may therefore be futile. Thus, we prefer to take the genetic determinants of human behavior (that, for example, we react strongly to visual stimuli) as givens, and to ask rather how those initial conditions shape individual and social learning [3]. Similar efforts have been undertaken by others, such as Henrich and Boyd [28] and Kendal et al. [20].

The sorts of models put forth by Cavalli-Sforza and Feldman, Boyd and Richerson, and others are a beginning towards the examination of a colossal problem. To such approaches, we must add efforts to understand ideation (how an idea for a behavior that becomes a norm gets invented in first place), and filtering (which ideas are accepted and which are rejected). How many ideas just pop up in someone's brain like a mutation? How many are slowly assembled from diverse data in a single mind? How many are the result of group “brainstorming?” How, for example, did an idea like the existence of an afterlife first get generated? Why do ideas spread, and what facilitates or limits that spread? What determines which ideas make it through transmission filters? Why are broadly held norms, like religious observance, most often not universal (why, for instance, has atheism always existed [29,30])? Ideas may be simply stated, or argued for, but transmission does not necessarily entail the reception or adoption of behaviors based on the idea, e.g., [31]. What we accept, and what gets stored in long-term memory, is but a tiny sample of a bombardment of candidate ideas, and understanding the nature and origin of filters is obviously one key to understanding the life spans of ideas and associated behaviors once generated.

## The Emergence of Higher-Level Structure: Some Simple Models

**Figure 1:**
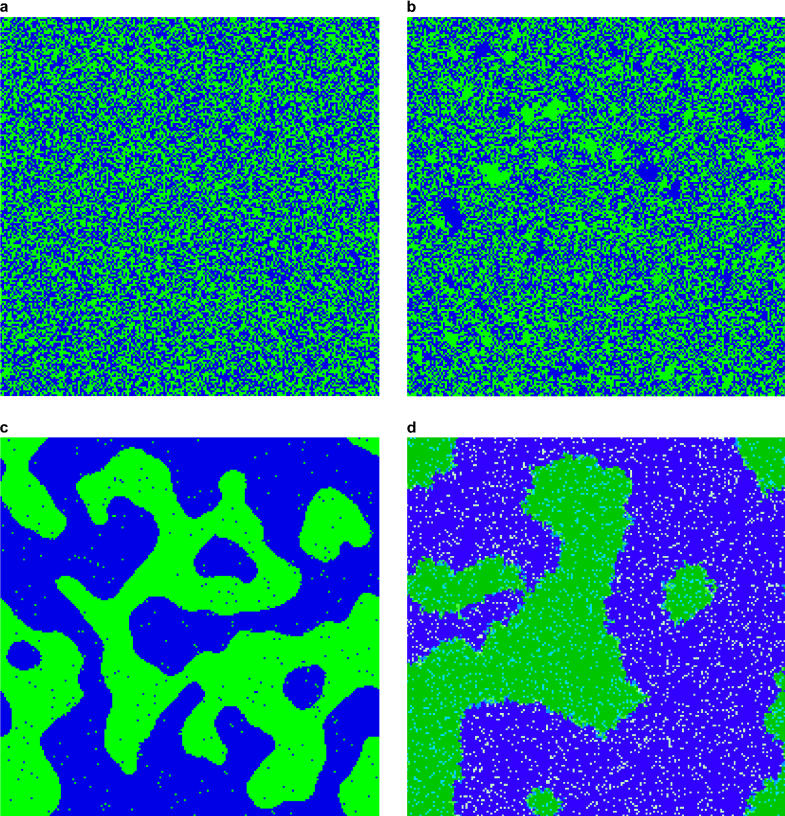
(A) Long-term patterning in the dynamics of two opinions for the threshold voter model with a low threshold. (B) Long-term patterning in the dynamics of two opinions for the threshold voter model with a high threshold. Note the existence of small, frozen clusters. (C) Long-term patterning in the dynamics of two opinions for the threshold voter model with an intermediate threshold. Note the clear emergence of group structure. (D) Long-term patterning in a model of social group formation, in which individuals imitate the opinions of others in their (two) groups, and others of similar opinions, and may switch groups when their views deviate from group norms.

Our filters usually are themselves products of cultural evolution, just as degrees of resistance of organisms to epidemics are products of genetic evolution. Filters include the perceived opinions of others, especially those viewed as members of the same self-defined social group, which collectively attempt to limit deviance [32–34]. “Conformist transmission,” defined as the tendency to imitate the most frequent behavior in the population, can help stabilize norms [28] and indeed can be the principal mechanism underlying the endogenous emergence of norms. The robustness of norms can arise either from the slow time scales on which group norms shift, or from the inherent resistance of individuals to changing their opinions. In the simplest exploration of this, Durrett and Levin (unpublished data) have examined the dynamics of the “threshold” voter model, in which individuals change their views if the proportion of neighbors with a different opinion exceeds a specified threshold. Where the threshold is low, individuals are continually changing their opinions, and groups cannot form (Figure 1A). In contrast, at high thresholds, stickiness is high—opinions rarely change—and the system quickly becomes frozen (Figure 1B). Again, groups cannot form. In between, however, at intermediate thresholds (pure conformist transmission), groups form and persist (Figure 1C). In the simplest such models in two dimensions, unanimity of opinions will eventually occur, but only over much longer time periods than those of group formation (see also [20]). When the possibility of innovation (mutation) is introduced in a model that considers linkages among traits and group labels, and where individuals can shift groups when their views deviate from group norms sufficiently, multiple opinions and multiple groups can persist, essentially, indefinitely (Figure 1D).

The formation of groups is the first step in the emergence of normative behavior; the work of Durrett and Levin shows that this can occur endogenously, caused by no more than a combination of ideation and imitation. The existence of a threshold helps to stabilize these groups, and to increase stickiness; furthermore, if threshold variation is permitted within populations, these thresholds can coevolve with group dynamics. What will the consequences be for the size distribution of groups, and for their persistence? Will group stability increase, while average size shrinks? What will be the consequences of allowing different individuals to have different thresholds, or of allowing everyone's thresholds to change with the size of the group? When payoffs reward individuals who adhere to group norms, and when individuals have different thresholds, will those thresholds evolve? The answers to such questions could provide deep insights into the mechanisms underlying the robustness of norms, and are ripe for investigation through such simple and transparent mathematical models.

Modeling may also shed light on why some norms (like fashions) change so easily, while others (like foot binding in imperial China) persist over centuries, and more generally on how tastes and practices evolve in societies. Norms in art and music change rapidly and with little apparent effort at persuasion or coercion. But three-quarters of a century of communism barely dented the religious beliefs of many Russians, despite draconian attempts to suppress them [35], and several centuries of science have apparently not affected the belief of a large number of Americans in angels and creationism (e.g., [36,37]). Then there are the near-universal norms, such as the rules against most types of physical assault or theft within groups that, although they vary in their specifics, are interpreted as necessary to preserve functional societies. Group-selection explanations for such phenomena (e.g., [12]) are, we argue, neither justified nor necessary (see also pp. 221–225 of [38], [39]). Such behaviors can emerge from individual-based models, simply involving rewards to individuals who belong to groups.

There are degrees: the evolution of cooperation is facilitated by tight interactions, for example when individuals interact primarily with their nearest neighbors [40,41], and the payoffs that come to individuals from such cooperation can enhance the tightness of interactions and the formation of groups. This easily explains why mutually destructive behaviors, like murder, are almost universally proscribed. Group benefits can emerge, and can enhance these effects, but it is neither necessary nor likely that group selection among groups for these behaviors overrides individual selection within groups when these groups are not composed of closely related individuals [42].

Simple models could address such things as the role of contagion in cultural evolution, recognized in one of the first works on psychology [43] in the context of religious revivals and belief, as what has been described as “pious contagion” (p. 10 of [30]). But models must also address issues such as the roles of authority or moral entrepreneurs (individuals engaged in changing a norm) [32], to say nothing of the impacts of advertising and the norm-changing efforts of the entertainment and other industries. In reality, we are intentioned agents who act with purpose. In maturing, we master the norms that have been evolved over a long period, but to which we may adapt in different ways and even (in the case of moral entrepreneurs) strive to change.

For a moral entrepreneur, a group that is too small may have little influence and be not worth joining. But large groups may be too difficult to influence, so also may not be worth joining. For such individuals, there is likely an optimal group size, depending on the change the individual wants to effect. Groups also introduce ancillary benefits of membership that change the equation. Such considerations influence decisions such as whether to join a third party effort in a political campaign; understanding the interplay between individual decisions and the dynamics of party sizes is a deeply important and fascinating question, with strong ecological analogies. Groups, collectively, must also wrestle with the costs and benefits of increasing membership, thereby enhancing influence while potentially diminishing consensus and hence the perceived benefits to members.

## Innovation and Conservatism

Cultural evolution, like biological evolution, contains what we like to call the “paradox of viscosity.” Evolving organisms must balance the need to change at an appropriate rate in response to varying environmental conditions against the need to maintain a functioning phenome. This trade-off between conservatism and adaptability, between stability and exploration, is one of the central problems in evolutionary theory. For example, how much change can there be in the genes required to maintain adaptation in a caterpillar without lethally affecting the structure and functioning of the butterfly (p. 303 of [44])? Conservatism in religion might be explained by the lack of empirical tests of religious ideas. But even in military technology and tactics, where empirical tests are superabundant, changes are slower than might be expected. For example, the British high command in World War I did not react rapidly to the realities of barbed wire, massed artillery, and machine guns [45]. Even so, the conservatism of the generals may be overrated [46].

## Macroscopic Dynamics

We have thus far examined the evolution of norms in isolation—as how the views of individuals (and thus the constituents of a pool of nongenetic information) change through time. But everywhere in common discourse and technical literature, it is assumed that norms are bundled into more or less discrete packages we call cultures, and that those packages themselves evolve. Recall that everyday notions such as that American culture of the 1990s was very different from that of the 1960s, that Islamic culture did not undergo the sort of reformation that convulsed Christian culture (for example, [47]), and that Alexander the Great carried Greek culture throughout the Mediterranean and as far east as Persia. The problem of defining “cultures” in cultural evolution seems analogous to that of defining “species” (or other categories) in genetic evolution. There has been a long and largely fruitless argument among taxonomists over the latter [48], and an equally fruitless debate in anthropology (and biology) on the definition of culture [39, 49–57].

Again, we suggest that the parsing of the various influences that create and sustain norms and cultures are ripe for theoretical modeling, but it must begin to incorporate the full richness on multiple scales of space, time, and complexity. Durrett and Levin [3] develop a model integrating the dynamics of clusters of linked opinions and group membership; appropriate extensions would allow group characteristics to evolve as well, but on slower time scales. The oversimplicity of models of symmetric imitation on regular grids, as represented in our simple models, must give way to those that incorporate fitnesses and feedbacks, as well as asymmetries and power brokers, on more complex networks of interaction [58].

## Challenges and Hypotheses

One of the major challenges for those interested in the evolution of norms is, at the most elementary level, defining a norm. This is related to another general problem of defining exactly what is changing in cultural evolution—which we might call the “meme dilemma” in honor of Dawkins' regrettably infertile notion. A second major challenge is discovering the mechanism(s) by which truly novel ideas and behaviors are generated and spread. A third is discovering the most effective ways of changing norms.

We've got a long way to go before being able to meet those challenges. One place to start is to begin formulating hypotheses about the evolution of norms that can be tested with historical data, modeling, or even (in some cases) experiments. Some hypotheses we believe worth testing (and some of which may well be rejected) are given in Box 1.

**Box 1. Sample Hypotheses about the Evolution of Norms**

**Hypothesis 1.** Evolution of technological norms will generally be more rapid than that of ethical norms.

Technological changes are generally tested promptly against environmental conditions—a round wheel wins against a hexagonal one every time, and the advantages of adopting it are clear to all. Ethical systems, on the other hand cannot often be tested against one another, and the standards of success are not only generally undetermined, they often vary from observer to observer and are the subject of ongoing controversy among philosophers.

**Hypothesis 2.** In societies with nonreligious art, the evolution of norms in art will be more rapid than those in religion.

We hypothesize that art is less important to the average individual than his or her basic system of relating to the world, and conservatism in the latter would be culturally adaptive (leading to success within a culture).

**Hypothesis 3.** Military norms will change more in defeated nations than victorious ones.

Was the Maginot Line and the generally disastrous performance of the French army in 1940 an example of a more general rule? Does success generally breed conservatism?

**Hypothesis 4.** The spread of a norm is not independent of the spread of others, but depends on the spread of other norms (norm clusters).

Does, for example, empathy decrease with social stratification?

**Hypothesis 5.** Susceptibility to the spread of norms is negatively correlated with level of education.

Are the less educated generally more conformist, or does the spread of norms depend almost entirely on the character of the norm?

**Hypothesis 6.** Horizontal transmission will show less stickiness than vertical transmission.

This conjecture is based on anecdotal observations that norms like using hula hoops come and go and are primarily horizontally transmitted, and religious values and other high-viscosity points of view are mostly vertically transmitted (p. 129 of [14], [59]).

In this essay we have tried to be provocative rather than exhaustive. There is a welter of issues we have not even attempted to address, including: (1) asymmetries of power in the spread of norms, (2) the role of networks, (3) the efficacy of persuasion as opposed to imitation, (4) the cause of thresholds in the change of norms, (5) the genesis of norms during child development, (6) the connection between attitudes and actions, (8) competition among norms from different cultures; and (9) the question, can norms exist “free of people” in institutions? Institutions certainly may emerge as independent structures, stabilized by laws and customs that are enforced to varying degrees through formal punishment or social pressure. Can such norms persist long even when adherence to them is disappearing? The interplay between the dynamics of individual behaviors and normative rules, operating on different time (and other) scales, may be the key, we argue, to understanding sudden phase transitions that can transform the cultural landscape.

We hope that, by being provocative, we can interest more evolutionists, behavioral biologists, and ecologists in tackling the daunting but crucial problems of cultural evolution. Few issues in science would seem to be more pressing if civilization is to survive.
